# 2-Acetyl­phenyl (2*E*)-3-(4-fluoro­phen­yl)acrylate

**DOI:** 10.1107/S1600536812040536

**Published:** 2012-09-29

**Authors:** Mehbub I. K. Momin, Sunayna Pawar, Neil Anthony Koorbanally, Hong Su, Deresh Ramjugernath

**Affiliations:** a School of Chemistry and Physics, University of KwaZulu-Natal, Private Bag X54001, Durban 4000, South Africa; b Chemistry Deparment, University of Cape Town, Rondebosch, 7701; c School of Engineering, University of KwaZulu-Natal, Private Bag X54001, Durban, South Africa

## Abstract

In the title compound, C_17_H_13_FO_3_, the dihedral angle between the benzene rings is 70.34 (5)°. In the crystal, molecules are linked *via* pairs of bifurcated C—H⋯(O,O) hydrogen bonds, forming inversion dimers. These dimers are linked *via* C—H⋯O and C—H⋯F inter­actions, forming a three-dimensional structure.

## Related literature

For the preparation, see: Pinto *et al.* (2000 ▶). For related structures, see: Santos *et al.* (2009 ▶); Ren, Li *et al.* (2006 ▶); Ren, Zhang *et al.* (2006 ▶). For bond-length data, see: Allen *et al.* (1987 ▶). The title compound is a core structure in various natural and pharmaceutically active compounds, displaying a broad spectrum of activity, see: Gomes *et al.* (2010 ▶).
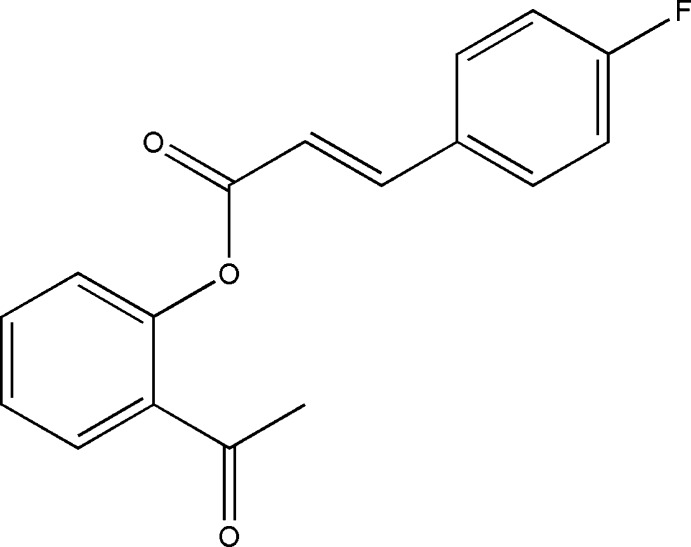


## Experimental

### 

#### Crystal data

C_17_H_13_FO_3_
*M* *_r_* = 284.27
Monoclinic, 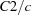

*a* = 26.574 (1) Å
*b* = 6.3883 (3) Å
*c* = 19.3304 (6) Å
β = 123.037 (2)°
*V* = 2751.01 (19) Å^3^
*Z* = 8
Mo *K*α radiation
μ = 0.10 mm^−1^
*T* = 173 K
0.26 × 0.23 × 0.09 mm

#### Data collection

Nonius Kappa CCD diffractometer
6005 measured reflections
3150 independent reflections
2201 reflections with *I* > 2σ(*I*)
*R*_int_ = 0.021

#### Refinement

*R*[*F* ^2^ > 2σ(*F* ^2^)] = 0.041
*wR*(*F* ^2^) = 0.115
*S* = 1.05
3150 reflections
191 parameters
H-atom parameters constrained
Δρ_max_ = 0.18 e Å^−3^
Δρ_min_ = −0.20 e Å^−3^

### 

Data collection: *COLLECT* program (Nonius, 2000 ▶); cell refinement: *DENZO-SMN* (Otwinowski & Minor, 1997 ▶); data reduction: *DENZO-SMN*; program(s) used to solve structure: *SHELXS97* (Sheldrick, 2008 ▶); program(s) used to refine structure: *SHELXL97* (Sheldrick, 2008 ▶); molecular graphics: *ORTEP-3* (Farrugia, 2012 ▶); software used to prepare material for publication: *WinGX* (Farrugia, 2012 ▶).

## Supplementary Material

Crystal structure: contains datablock(s) I, global. DOI: 10.1107/S1600536812040536/fj2596sup1.cif

Structure factors: contains datablock(s) I. DOI: 10.1107/S1600536812040536/fj2596Isup2.hkl

Supplementary material file. DOI: 10.1107/S1600536812040536/fj2596Isup3.cml

Additional supplementary materials:  crystallographic information; 3D view; checkCIF report

## Figures and Tables

**Table 1:** Hydrogen-bond geometry (Å, °) *Cg*1 is the centroid of the C3–C8 ring.

*D*—H⋯*A*	*D*—H	H⋯*A*	*D*⋯*A*	*D*—H⋯*A*
C7—H7⋯F1^i^	0.95	2.52	3.2402 (16)	132
C11—H11⋯O3^ii^	0.95	2.46	3.3369 (16)	154
C13—H13⋯O3^ii^	0.95	2.45	3.3191 (16)	153
C16—H16⋯O1^iii^	0.95	2.51	3.3590 (17)	149
C6—H6⋯*Cg*1^iv^	0.95	2.99	3.818 (1)	146

Symmetry codes: (i) 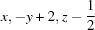; (ii) 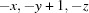; (iii) 
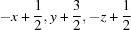
; (iv) 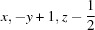.

**Fig. 1.**
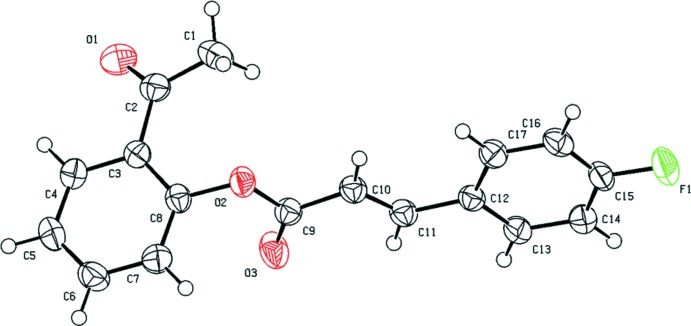
ORTEP diagram showing the molecular structure of the titled compound with atomic labelling scheme. Non-H atoms are drawn with 50% probability displacement ellipsoids and H atoms are shown as open circles.

## supplementary crystallographic information

### Comment

The title compound (*E*)-2-acetylphenyl-3-(4-fluorophenyl) acrylate was obtained as an intermediate en route to the synthesis of 4'-fluoro-2-styrylchromone and easily converts to the 2-hydroxyphenyl pentadienone with DMSO in the presence of a strong base (Santos *et al.*, 2009). It was synthesized according to the procedure by Pinto *et al.* (2000) with modification. The title compound is a core structure in various natural and pharmaceutically active compounds, displaying a broad spectrum of activity (Gomes *et al.*, 2010).

In the molecule of the title compound (Fig. 1), the two aromatic rings (ring 1: C3—C4—C5—C6—C7—C8; ring 2: C12—C13—C14—C15—C16—C17—C18) are almost perpendicular to each other with a dihedral angle of 70.34 (5)°. The torsion angle C9—C10—C11—C12 is -178.8 (1)*^o^*, indicating a *trans* configuration of the double bond. All bond lengths and angles are within normal ranges (Allen *et al.*,1987). In the crytsal packing, ring 1 adopts a parallel offset arrangement with itself of the neighbouring molecule with centroidal distance of 4.125 (1) Å. The crystal is further stablized by a number of weak hydrogen bonds with the type C—H···*X* (*X* = O or F) and C—H···π (Table 1).

### Experimental

Phosphorous oxychloride (15.6 mmol) was added to a solution of 2-hydroxyacetophenone (12.0 mmol) and 4'-fluoro cinnamic acid (15.6 mmol) in dry pyridine. The solution was stirred at 60–70 °C for 3 h, then poured into ice and water and the reaction mixture acidified with hydrochloric acid (pH 3–4). The obtained solid was removed by filtration and dissolved in ethyl acetate (100 ml) and purified by silica gel column chromatography using a 7:3 mixture of ethyl acetate:n-hexane as the eluent. The solvent was evaporated to dryness and the residue recrystallized from ethanol, resulting in the title compound with a 72% yield and a m.p of 80–82°C.

IR (KBr) ν_max_ (cm^-1^): 1729 (C=O), 1670 (C=O), 1624 (C=C), 1590, 1446, 1221 (C—F), 1202, 1159, 1050. ^1^H NMR (CDCl_3_, 400 MHz): δ 7.84 (d, *J* = 15.96 Hz, 1H, Hβ), 7.81 (dd, *J* = 7.56,1.60 Hz, 1H), 7.58 (dd, *J* = 8.60, 5.44, 2H), 7.54 (ddd, *J* = 8.04, 7.88, 1.60 Hz, 1H), 7.33 (ddd, *J* = 8.04, 7.56, 0.72 Hz, 1H), 7.17 (d, *J* = 8.04 Hz, 1H), 7.09 (t, *J* = 8.60 Hz, 2H), 6.58 (d, *J* = 15.96 Hz, 1H, Hα), 2.54 (s, 3H, CH_3_). ^13^C NMR (CDCl_3_, 100 MHz): δ 197.78 (C=O), 165.14 (C=O), 164.25 (d, *J*_CF_ = 250.70 Hz), 149.07, 145.99, 133.36, 131.30, 130.43, 130.24 ((d, *J*_CF_ = 19.46 Hz), 130.15, 126.10, 123.78, 116.58, 116.20 (d, *J*_CF_ = 21.85 Hz), 29.71 (CH_3_). ^19^F NMR (CDCl_3_, 376.5 MHz): δ -108.54. EIMS (probe) 70 eV (*m/z*, *rel*. int.) 284 (*M*^+^) (21.42), 149 (100), 121 (25), 101 (20).

### Refinement

All non-hydrogen atoms were refined anisotropically. All hydrogen atoms could be found in the difference electron density maps but were finally placed in idealized positions refining in riding models with *U*_iso_ set at 1.2 or 1.5 times *U*_eq_ of their parent atoms.

### Crystal data

**Table:**

C_17_H_13_FO_3_	*F*(000) = 1184
*M**_r_* = 284.27	*D*_x_ = 1.373 Mg m^−^^3^
Monoclinic, *C*2/*c*	Mo *K*α radiation, λ = 0.71073 Å
Hall symbol: -C 2yc	Cell parameters from 6005 reflections
*a* = 26.574 (1) Å	θ = 3.1–27.5°
*b* = 6.3883 (3) Å	µ = 0.10 mm^−^^1^
*c* = 19.3304 (6) Å	*T* = 173 K
β = 123.037 (2)°	Plate, colourless
*V* = 2751.01 (19) Å^3^	0.26 × 0.23 × 0.09 mm
*Z* = 8	

### Data collection

**Table:**

Nonius Kappa CCD diffractometer	2201 reflections with *I* > 2σ(*I*)
Radiation source: fine-focus sealed tube	*R*_int_ = 0.021
Graphite monochromator	θ_max_ = 27.5°, θ_min_ = 3.1°
1.2° φ scans and ω scans	*h* = −33→34
6005 measured reflections	*k* = −8→8
3150 independent reflections	*l* = −25→24

### Refinement

**Table:**

Refinement on *F*^2^	Primary atom site location: structure-invariant direct methods
Least-squares matrix: full	Secondary atom site location: difference Fourier map
*R*[*F*^2^ > 2σ(*F*^2^)] = 0.041	Hydrogen site location: inferred from neighbouring sites
*wR*(*F*^2^) = 0.115	H-atom parameters constrained
*S* = 1.05	*w* = 1/[σ^2^(*F*_o_^2^) + (0.0612*P*)^2^ + 0.6743*P*] where *P* = (*F*_o_^2^ + 2*F*_c_^2^)/3
3150 reflections	(Δ/σ)_max_ < 0.001
191 parameters	Δρ_max_ = 0.18 e Å^−^^3^
0 restraints	Δρ_min_ = −0.20 e Å^−^^3^

### Special details

**Table:**

Geometry. All e.s.d.'s (except the e.s.d. in the dihedral angle between two l.s. planes) are estimated using the full covariance matrix. The cell e.s.d.'s are taken into account individually in the estimation of e.s.d.'s in distances, angles and torsion angles; correlations between e.s.d.'s in cell parameters are only used when they are defined by crystal symmetry. An approximate (isotropic) treatment of cell e.s.d.'s is used for estimating e.s.d.'s involving l.s. planes.
Refinement. Refinement of *F*^2^ against ALL reflections. The weighted *R*-factor *wR* and goodness of fit *S* are based on *F*^2^, conventional *R*-factors *R* are based on *F*, with *F* set to zero for negative *F*^2^. The threshold expression of *F*^2^ > σ(*F*^2^) is used only for calculating *R*-factors(gt) *etc*. and is not relevant to the choice of reflections for refinement. *R*-factors based on *F*^2^ are statistically about twice as large as those based on *F*, and *R*- factors based on ALL data will be even larger.

### Fractional atomic coordinates and isotropic or equivalent isotropic displacement parameters (Å^2^)

**Table:**

	*x*	*y*	*z*	*U*_iso_*/*U*_eq_	
F1	0.04188 (4)	1.49324 (13)	0.22111 (6)	0.0580 (3)	
O1	0.26072 (5)	−0.02536 (18)	0.16114 (6)	0.0556 (3)	
O2	0.16132 (4)	0.49823 (13)	0.02908 (6)	0.0379 (2)	
O3	0.06857 (4)	0.41188 (17)	−0.00442 (7)	0.0522 (3)	
C1	0.25590 (7)	0.3344 (3)	0.18081 (9)	0.0512 (4)	
H1A	0.2225	0.3695	0.1867	0.077*	
H1B	0.2633	0.4515	0.1548	0.077*	
H1C	0.2921	0.3074	0.2354	0.077*	
C2	0.24038 (6)	0.1439 (2)	0.12848 (8)	0.0377 (3)	
C3	0.19990 (5)	0.1519 (2)	0.03588 (7)	0.0315 (3)	
C4	0.19941 (6)	−0.0253 (2)	−0.00725 (8)	0.0369 (3)	
H4	0.2241	−0.1415	0.0228	0.044*	
C5	0.16398 (6)	−0.0351 (2)	−0.09234 (9)	0.0430 (4)	
H5	0.1643	−0.1569	−0.1203	0.052*	
C6	0.12814 (7)	0.1330 (2)	−0.13652 (8)	0.0447 (4)	
H6	0.1039	0.1274	−0.1951	0.054*	
C7	0.12732 (6)	0.3091 (2)	−0.09597 (8)	0.0418 (3)	
H7	0.1026	0.4247	−0.1265	0.050*	
C8	0.16273 (6)	0.3169 (2)	−0.01042 (8)	0.0327 (3)	
C9	0.11003 (6)	0.5312 (2)	0.02865 (8)	0.0337 (3)	
C10	0.11450 (6)	0.72268 (19)	0.07306 (8)	0.0339 (3)	
H10	0.1496	0.8069	0.0972	0.041*	
C11	0.06885 (6)	0.7778 (2)	0.07935 (8)	0.0346 (3)	
H11	0.0355	0.6853	0.0548	0.042*	
C12	0.06392 (6)	0.96377 (19)	0.11951 (7)	0.0321 (3)	
C13	0.01102 (6)	0.9974 (2)	0.11673 (8)	0.0379 (3)	
H13	−0.0204	0.8971	0.0906	0.045*	
C14	0.00350 (6)	1.1745 (2)	0.15135 (8)	0.0418 (3)	
H14	−0.0326	1.1971	0.1493	0.050*	
C15	0.04946 (6)	1.3164 (2)	0.18860 (8)	0.0388 (3)	
C16	0.10298 (6)	1.2888 (2)	0.19487 (8)	0.0385 (3)	
H16	0.1344	1.3885	0.2226	0.046*	
C17	0.10980 (6)	1.1118 (2)	0.15972 (8)	0.0360 (3)	
H17	0.1464	1.0903	0.1629	0.043*	

### Atomic displacement parameters (Å^2^)

**Table:**

	*U*^11^	*U*^22^	*U*^33^	*U*^12^	*U*^13^	*U*^23^
F1	0.0663 (6)	0.0476 (5)	0.0611 (6)	−0.0033 (4)	0.0354 (5)	−0.0236 (4)
O1	0.0560 (7)	0.0582 (7)	0.0411 (6)	0.0116 (5)	0.0191 (5)	0.0104 (5)
O2	0.0366 (5)	0.0330 (5)	0.0456 (5)	−0.0029 (4)	0.0234 (4)	−0.0093 (4)
O3	0.0453 (6)	0.0485 (6)	0.0693 (7)	−0.0152 (5)	0.0354 (6)	−0.0258 (5)
C1	0.0492 (9)	0.0627 (10)	0.0346 (8)	−0.0076 (7)	0.0183 (7)	−0.0089 (7)
C2	0.0314 (7)	0.0486 (8)	0.0358 (7)	−0.0011 (6)	0.0201 (6)	−0.0001 (6)
C3	0.0303 (6)	0.0351 (7)	0.0331 (7)	−0.0026 (5)	0.0200 (5)	−0.0011 (5)
C4	0.0397 (7)	0.0348 (7)	0.0413 (8)	0.0037 (6)	0.0254 (6)	0.0013 (6)
C5	0.0512 (9)	0.0416 (8)	0.0440 (8)	−0.0030 (7)	0.0311 (7)	−0.0103 (7)
C6	0.0468 (8)	0.0541 (9)	0.0317 (7)	−0.0014 (7)	0.0205 (6)	−0.0045 (7)
C7	0.0437 (8)	0.0426 (8)	0.0361 (7)	0.0060 (6)	0.0199 (6)	0.0037 (6)
C8	0.0346 (7)	0.0305 (7)	0.0366 (7)	−0.0031 (5)	0.0217 (6)	−0.0039 (5)
C9	0.0348 (7)	0.0327 (7)	0.0334 (7)	0.0005 (6)	0.0183 (6)	0.0001 (5)
C10	0.0355 (7)	0.0296 (7)	0.0341 (7)	−0.0025 (5)	0.0174 (6)	−0.0019 (5)
C11	0.0352 (7)	0.0305 (7)	0.0344 (7)	−0.0032 (5)	0.0165 (6)	−0.0030 (5)
C12	0.0352 (7)	0.0295 (7)	0.0283 (6)	0.0002 (5)	0.0151 (5)	−0.0001 (5)
C13	0.0340 (7)	0.0377 (7)	0.0372 (7)	−0.0044 (6)	0.0164 (6)	−0.0086 (6)
C14	0.0366 (7)	0.0463 (8)	0.0408 (8)	0.0010 (6)	0.0199 (6)	−0.0080 (6)
C15	0.0492 (8)	0.0322 (7)	0.0313 (7)	0.0017 (6)	0.0196 (6)	−0.0063 (6)
C16	0.0424 (8)	0.0332 (7)	0.0343 (7)	−0.0080 (6)	0.0172 (6)	−0.0048 (6)
C17	0.0377 (7)	0.0338 (7)	0.0364 (7)	−0.0026 (6)	0.0201 (6)	−0.0015 (6)

### Geometric parameters (Å, º)

**Table:**

F1—C15	1.3600 (15)	C7—C8	1.3878 (18)
O1—C2	1.2195 (17)	C7—H7	0.9500
O2—C9	1.3748 (16)	C9—C10	1.4614 (17)
O2—C8	1.3993 (15)	C10—C11	1.3310 (19)
O3—C9	1.1982 (16)	C10—H10	0.9500
C1—C2	1.490 (2)	C11—C12	1.4636 (17)
C1—H1A	0.9800	C11—H11	0.9500
C1—H1B	0.9800	C12—C13	1.3937 (18)
C1—H1C	0.9800	C12—C17	1.3972 (18)
C2—C3	1.5048 (19)	C13—C14	1.3835 (18)
C3—C8	1.3869 (18)	C13—H13	0.9500
C3—C4	1.4014 (18)	C14—C15	1.369 (2)
C4—C5	1.381 (2)	C14—H14	0.9500
C4—H4	0.9500	C15—C16	1.371 (2)
C5—C6	1.379 (2)	C16—C17	1.3814 (19)
C5—H5	0.9500	C16—H16	0.9500
C6—C7	1.378 (2)	C17—H17	0.9500
C6—H6	0.9500		
			
C9—O2—C8	116.42 (9)	O3—C9—O2	121.72 (12)
C2—C1—H1A	109.5	O3—C9—C10	127.06 (12)
C2—C1—H1B	109.5	O2—C9—C10	111.22 (11)
H1A—C1—H1B	109.5	C11—C10—C9	119.26 (12)
C2—C1—H1C	109.5	C11—C10—H10	120.4
H1A—C1—H1C	109.5	C9—C10—H10	120.4
H1B—C1—H1C	109.5	C10—C11—C12	127.80 (12)
O1—C2—C1	119.47 (12)	C10—C11—H11	116.1
O1—C2—C3	118.28 (12)	C12—C11—H11	116.1
C1—C2—C3	122.25 (12)	C13—C12—C17	118.24 (12)
C8—C3—C4	117.21 (11)	C13—C12—C11	119.00 (11)
C8—C3—C2	126.11 (11)	C17—C12—C11	122.76 (12)
C4—C3—C2	116.67 (11)	C14—C13—C12	121.07 (12)
C5—C4—C3	121.63 (13)	C14—C13—H13	119.5
C5—C4—H4	119.2	C12—C13—H13	119.5
C3—C4—H4	119.2	C15—C14—C13	118.25 (13)
C6—C5—C4	119.62 (13)	C15—C14—H14	120.9
C6—C5—H5	120.2	C13—C14—H14	120.9
C4—C5—H5	120.2	F1—C15—C14	118.60 (13)
C7—C6—C5	120.20 (12)	F1—C15—C16	118.28 (12)
C7—C6—H6	119.9	C14—C15—C16	123.12 (12)
C5—C6—H6	119.9	C15—C16—C17	118.02 (12)
C6—C7—C8	119.76 (13)	C15—C16—H16	121.0
C6—C7—H7	120.1	C17—C16—H16	121.0
C8—C7—H7	120.1	C16—C17—C12	121.26 (12)
C3—C8—C7	121.56 (12)	C16—C17—H17	119.4
C3—C8—O2	119.91 (11)	C12—C17—H17	119.4
C7—C8—O2	118.51 (11)		
			
O1—C2—C3—C8	164.97 (13)	C8—O2—C9—O3	−0.64 (18)
C1—C2—C3—C8	−14.7 (2)	C8—O2—C9—C10	178.87 (10)
O1—C2—C3—C4	−14.28 (18)	O3—C9—C10—C11	0.0 (2)
C1—C2—C3—C4	166.04 (12)	O2—C9—C10—C11	−179.43 (12)
C8—C3—C4—C5	0.71 (19)	C9—C10—C11—C12	−178.78 (11)
C2—C3—C4—C5	−179.97 (12)	C10—C11—C12—C13	177.62 (13)
C3—C4—C5—C6	0.1 (2)	C10—C11—C12—C17	−1.4 (2)
C4—C5—C6—C7	−0.5 (2)	C17—C12—C13—C14	1.26 (19)
C5—C6—C7—C8	0.0 (2)	C11—C12—C13—C14	−177.78 (12)
C4—C3—C8—C7	−1.21 (18)	C12—C13—C14—C15	0.0 (2)
C2—C3—C8—C7	179.54 (12)	C13—C14—C15—F1	178.52 (11)
C4—C3—C8—O2	−179.87 (11)	C13—C14—C15—C16	−1.6 (2)
C2—C3—C8—O2	0.88 (19)	F1—C15—C16—C17	−178.23 (11)
C6—C7—C8—C3	0.9 (2)	C14—C15—C16—C17	1.9 (2)
C6—C7—C8—O2	179.56 (12)	C15—C16—C17—C12	−0.56 (19)
C9—O2—C8—C3	−109.06 (13)	C13—C12—C17—C16	−0.95 (19)
C9—O2—C8—C7	72.24 (15)	C11—C12—C17—C16	178.05 (12)

### Hydrogen-bond geometry (Å, º)

Cg1 is the centroid of the C3–C8 ring.

**Table:**

*D*—H···*A*	*D*—H	H···*A*	*D*···*A*	*D*—H···*A*
C1—H1*B*···O2	0.98	2.48	2.8245 (18)	100
C7—H7···F1^i^	0.95	2.52	3.2402 (16)	132
C11—H11···O3	0.95	2.50	2.8415 (16)	101
C11—H11···O3^ii^	0.95	2.46	3.3369 (16)	154
C13—H13···O3^ii^	0.95	2.45	3.3191 (16)	153
C16—H16···O1^iii^	0.95	2.51	3.3590 (17)	149
C6—H6···*Cg*1^iv^	0.95	2.99	3.818 (1)	146

Symmetry codes: (i) *x*, −*y*+2, *z*−1/2; (ii) −*x*, −*y*+1, −*z*; (iii) −*x*+1/2, *y*+3/2, −*z*+1/2; (iv) *x*, −*y*+1, *z*−1/2.

## References

[bb1] Allen, F. H., Kennard, O., Watson, D. G., Brammer, L., Orpen, A. G. & Taylor, R. (1987). *J. Chem. Soc. Perkin Trans. 2*, pp. S1–19.

[bb2] Farrugia, L. J. (2012). *J. Appl. Cryst.* **45**, 849–854.

[bb3] Gomes, A., Freitas, M., Fernandes, E. & Lima, J. L. F. C. (2010). *Mini Rev. Med. Chem.* **10**, 1–7. 10.2174/138955710791112550 20380638

[bb4] Nonius (2000). *COLLECT* Nonius BV, Delft, The Netherlands.

[bb5] Otwinowski, Z. & Minor, W. (1997). *Methods in Enzymology*, Vol. 276, *Macromolecular Crystallography*, Part A, edited by C. W. Carter Jr & R. M. Sweet, pp. 307–326. New York: Academic Press.

[bb6] Pinto, D. C. G. A., Silva, A. M. S. & Cavaleiro, J. A. S. (2000). *New J. Chem.* **24**, 85–92.

[bb7] Ren, R., Li, X.-M., Li, Q. & Zhang, S.-S. (2006). *Acta Cryst.* E**62**, o293–o294.

[bb8] Ren, R., Zhang, S.-S., Li, Q., Li, X.-M. & Song, X.-Y. (2006). *Acta Cryst.* E**62**, o160–o161.

[bb9] Santos, C. M. M., Silva, A. M. S. & Cavaleiro, J. A. S. (2009). *Eur. J. Org. Chem.* pp. 2642–2660.

[bb10] Sheldrick, G. M. (2008). *Acta Cryst.* A**64**, 112–122. 10.1107/S0108767307043930 18156677

